# Case of Triple Fracture of the Lower Jaw

**Published:** 1871-05

**Authors:** T. Curtis Smith


### SELECTED ARTICLES.

### ARTICLE V.

### *Case of Triple Fracture of the Lower Jaw.*

By T. Curtis Smith, M. D.

On the 5th of Sept., 1865, I was called to see Mr. G., æt. 69; farmer; healthy; of steady good habits, and considering his age, robust. Six days previous to my first visit, while grinding cane, the lever to which the horse was attached caught against the left side of his head, on a line corresponding with the inferior margin of the jaw and point of the mastoid process of the temporal bone. The right side of his head was carried against the sharp corner of the solid frame work of the grinder, on a line corresponding exactly with the one on the left side. As the lever passed over his head, it produced fracture of the inferior maxilla at the symphysis, and a fracture in each lateral half, about eight lines anterior to the angles of the jaw. It also tore off the left ear and the integument and fascia to the margin of the calvaria. It was seen the same day by a physician in good standing, and of many years experience, who pronounced it a fatal injury, he diagnosing "fracture of the skull." Left it without treatment of any description. Hence, the long period—six days—that elapsed before any treatment was instituted. I cleansed the wound, drew down the integument, and ordered free and frequent applications of cold water to it. Found the jaw fractured as above described, but not in the least displaced. It retained its natural situation, the fragments being in perfect apposition as long as he did not talk or attempt to eat. I put on a dressing to steady the fragments —Barton's dressing,—but it was painful and heating, the temperature of the atmosphere being very high. He would not allow it to remain. Perfect union of fragments occurred without the slightest deformity, and practically without the sign of a dressing. At the symphysis it was not solid until after four months, but near the lateral angles solid union had taken place in six weeks. He was kept on slops and fluid diet for the first eight weeks. I report the case on account of its being a triple fracture, and not recognizable by any deformity as long as the mouth was kept closed; but more especially on account of such ready union without any apparatus and without deformity, and that too in an individual 69 years of age. The other injuries healed kindly, leaving off, of course, the whole external left ear, but not injuring the hearing.

Our authorities report some cases of double and triple fractures of the lower jaw. Hamilton reports eleven cases of double and triple fractures. Of these, three were triple fractures, two of them resembling my own case. He also reports the case of an "Irish laborer" æt. 17 years, who was thrown from a wagon, breaking the inferior maxilla on both sides through the body, and also, exactly in the centre, vertically between the central incisors." With this case he seem to have had great difficulty in securing and retaining the fragments in perfect apposition. He also mentions a case recorded by Howzelot, "in which a fall from a height produced at the same time, a fracture of both condyles, of both coronoid processes, and of the symphysis," making five fractures in the same maxilla. In my case the symphasis frac. ture was vertical, the two lateral oblique. The fragments were evidently held in apposition by the muscular attachments being such as to balance the fragments equally.—*Med. and Surg. Reporter.*

---

